# Reliable Comparison of Pnicogen, Chalcogen, and Halogen Bonds in Complexes of 6-OXF_2_-Fulvene (X = As, Sb, Se, Te, Be, I) With Three Electron Donors

**DOI:** 10.3389/fchem.2020.608486

**Published:** 2020-12-09

**Authors:** Na Liu, Qingzhong Li, Sean A. C. McDowell

**Affiliations:** ^1^The Laboratory of Theoretical and Computational Chemistry, School of Chemistry and Chemical Engineering, Yantai University, Yantai, China; ^2^Department of Biological and Chemical Sciences, The University of the West Indies, Cave Hill Campus, Cave Hill, Barbados

**Keywords:** halogen bond, pnicogen bond, NBO, AIM, chalcogen bond

## Abstract

The pnicogen, chalcogen, and halogen bonds between 6-OXF_2_-fulvene (X = As, Sb, Se, Te, Br, and I) and three nitrogen-containing bases (FCN, HCN, and NH_3_) are compared. For each nitrogen base, the halogen bond is strongest, followed by the pnicogen bond, and the chalcogen bond is weakest. For each type of bond, the binding increases in the FCN < HCN < NH_3_ pattern. Both FCN and HCN engage in a bond with comparable strengths and the interaction energies of most bonds are < −6 kcal/mol. However, the strongest base NH_3_ forms a much more stable complex, particularly for the halogen bond with the interaction energy going up to −18 kcal/mol. For the same type of interaction, its strength increases as the mass of the central X atom increases. These bonds are different in strength, but all of them are dominated by the electrostatic interaction, with the polarization contribution important for the stronger interaction. The presence of these bonds changes the geometries of 6-OXF_2_-fulvene, particularly for the halogen bond formed by NH_3_, where the F-X-F arrangement is almost vertical to the fulvene ring.

## Introduction

Intermolecular interactions can regulate many chemical and biological processes (Oshovsky et al., 2007; Schneider, 2009; Zayed et al., 2010). Insight into these interactions is helpful in promoting development of supramolecular chemistry (Smith, 2005; Uhlenheuer et al., 2010; Bauzá et al., 2014), materials science (Müller-Dethlefs and Hobza, 2000; Vickaryous et al., 2004), and the rational design of new drugs and biochemistry (Xu et al., 2011; Lu et al., 2012). Among intermolecular interactions, the hydrogen bond (HB) is still widely investigated because of its importance in aqueous and biological systems. Of course, interest in other intermolecular interactions has been growing rapidly. These new types of intermolecular interactions were named for the type of atom that replaces the bridging proton in the HB, and these atoms include aerogen (Bauzá and Frontera, 2015a,b,c), halogen (Desiraju et al., 2013; Cavallo et al., 2016; Terraneo and Resnati, 2017), chalcogen (Azofra et al., 2015; Nziko and Scheiner, 2015; Nayak et al., 2017), pnicogen (Bauzá et al., 2016; Joy et al., 2016; Sánchez-Sanz et al., 2016), and tetrel (Grabowski, 2014a; Mani and Arunan, 2014; Li et al., 2015) atoms. Scientists have tried to provide a consistent explanation for the origin of these different intermolecular interactions. By means of molecular electrostatic potential (MEP) analyses, it was found that the surfaces of these covalently-bonded atoms all have positive MEPs (holes). Thus, these holes produce an attractive force with an approaching nucleophile. Usually, this hole is called σ-hole if this positive MEP region is located at the end of the covalent bond (Murray et al., 2007). On the other hand, the stability of all these bonds depends in part upon charge transfer from the electron-donor atom into an antibonding orbital of the acceptor (Scheiner, 2013a).

In studying these new intermolecular interactions, it is natural to compare them with HBs. Besides the consistency in the formation mechanism, they have similar applications in crystal materials (Gilday et al., 2015; Wang et al., 2016; Scilabra et al., 2019), molecular recognition (Cavallo et al., 2010; Ariga et al., 2012; Wenzel et al., 2012), chemical reactions (Walter et al., 2011; Liu et al., 2016; Wonner et al., 2017), and biological systems (Parisini et al., 2011; Lange et al., 2015; García-LLinás et al., 2017). For example, Scheiner compared the ability of the HB, halogen bond (XB), and tetrel bond (TB) in recognizing halides and found that TBs may be an effective halide receptor which displays good selectivity for both F^−^ and Cl^−^ anions (Scheiner, 2017). A systematic comparison of the angular geometries for B···ClF XB and B···HCl HB, as B is varied, was performed by Legon and parallels between angular geometries were observed for both XB and HB; the author claimed that the empirical rules for predicting angular geometries of HB can be extended to XB (Legon, 2008). Scheiner made a detailed comparison of the pnicogen bond (ZB) with the chalcogen bond (ChB), XB, and HB with regards to energetics, geometries, electron density shifts, and energy decomposition, and concluded that ZB has certain parallels with other bonds (Scheiner, 2013b). On the other hand, some differences were found among these bonds. Because pnicogen, chalcogen, and halogen atoms are strongly anisotropic, ZB, ChB, and XB are more sensitive to angular distortions than HB (Adhikari and Scheiner, 2012). Unlike HB and XB, the tetrel and pnicogen donor molecules are often distorted in order to accommodate the approaching base (Zierkiewicz et al., 2018).

The applications of intermolecular interactions are largely based upon their strengths; consequently, more attention was focused on their strengths and the competition between these bonds (Alkorta et al., 2008; Li et al., 2010; Solimannejad et al., 2012; An et al., 2013; Nagels et al., 2014; Guo et al., 2015; Dong et al., 2018; Lee et al., 2019). Hypohalous acids engage in a stronger HB, but a weaker XB, with various bases such as formaldehyde (Li et al., 2010), formamidine (An et al., 2013), NH_3_ and HCN (Alkorta et al., 2008). Dong et al. (2018) assessed the relative strengths of TB, ZB, ChB, and XB, which are represented by the third-row atoms Ge, As, Se, and Br, respectively. The H atoms around these central atoms were substituted firstly by a methyl group and then by F substitution in various locations (Dong et al., 2018). In the context of unsubstituted acids, the strengths of these bonds vary in the order ChB > XB > ZB ≈ TB, while F substitution at a position directly opposite the base results in a XB > ChB > ZB > TB ordering (Dong et al., 2018). In comparing ZB, ChB, and XB, the lone pairs around the central atoms are respectively three, two, and one. It is known that substituents have an important effect on the strength of these non-covalent bonds (Geboes et al., 2017). It is therefore necessary to bind equivalent atoms/groups to these pnicogen, chalcogen, and halogen atoms when comparing their strengths.

Fulvene is a non-aromatic molecule, but its aromaticity can be realized by substituents *exo* (6-position) to the five-membered ring (Krygowski et al., 2010). Fulvene and its derivatives, and particularly this 6-position substitution, have been studied due to their function in synthesis (Stone and Little, 1984; Strohfeldt and Tacke, 2008; Peloquin et al., 2012). The acidity of 6-OH-fulvene can be greatly increased by strong electron-withdrawing groups adjoined to the fulvene ring (Maksić and Vianello, 2004), which prompted us to explore the intermolecular interactions of the substituent at the 6-position of fulvene (Hou et al., 2019a,b). The presence of the fulvene skeleton strengthens the carbon bonding between 6-OCH_3_-fulvene and NH_3_ since its interaction energy is double that of the complex of methanol and NH_3_ (Hou et al., 2019a). The relative strengths of various bonds are also related to the nature and basicity of the base. Thioformaldehyde can reduce the difference in the binding strength between XB and HB in the HOBr complex (Li et al., 2011). If TeH_2_ acts as the electron donor, the XB is more favorable than the HB in complexes with 6-OX-fulvene (X = H, Cl, Br, I) (Hou et al., 2019b). This shows that introduction of a fulvene skeleton to an atom/group may cause an interesting result when this atom/group participates in an intermolecular interaction.

In this work, 6-OXF_2_-fulvene (X = As, Sb, Se, Te, Be, I) was the Lewis acid chosen to compare the strengths of ZB, ChB, and XB. All pnicogen, chalcogen, halogen atoms have the same number of substituents in their molecules. Moreover, these substituents are also the same for different X atoms. Thus, this molecule allows for a reliable comparison of these interactions since only X is varied. If X is a halogen atom, the corresponding 6-OXF_2_-fulvene molecule contains a hypervalent halogen. Hypervalent halogens have been shown to form a XB with various electron donors (Grabowski, 2014b; 2017; Kirshenboim and Kozuch, 2016). For X = Se and Te, there is similar bonding environment (Nordheider et al., 2015). Although 6-OXF_2_-fulvene (X = Se and Te) is neutral, it is a doublet, which is different from the halogen and pnicogen analogs. The electron donors selected range in electron-donating ability from FCN to HCN to NH_3_. The electron donors PH_3_ and AsH_3_ are compared with NH_3_. These complexes are also compared with the ZB, ChB, and XB of more conventional molecules found in the literature. We explore their similarities and differences in the present study by means of natural bond orbital (NBO), atoms in molecules (AIM), MEP, and energy decomposition (ED) analyses.

## Computational Methods

All calculations were performed using the Gaussian 09 suite of programs (Frisch et al., 2009). Geometries were optimized at the MP2 computational level with the aug-cc-pVDZ basis set for all atoms except I, Sb, and Te, for which the aug-cc-pVDZ-PP basis set with relativistic corrections was adopted. Vibrational frequency calculations at the same level confirmed that the structures obtained correspond to energetic minima. The interaction energy was calculated by the supermolecular method involving the energies of the monomers at the geometries they adopt within the complex. This quantity was corrected for the basis set superposition error (BSSE) by the counterpoise protocol proposed by Boys and Bernardi (1970). The interaction energy was also decomposed into five terms including electrostatic, exchange, repulsion, polarization, and dispersion energies using the GAMESS program (Schmidt et al., 1993) and localized molecular orbital-energy decomposition analysis (LMOEDA) method (Su and Li, 2009) at the MP2/aug-cc-pVDZ(PP) level. Using the natural bond orbital (NBO) method (Reed et al., 1988) implemented in the Gaussian 09 program, the charge transfer and second-order perturbation energies were obtained. The AIM2000 package (Bader, 1990) was used to assess the topological parameters at each bond critical point (BCP), including the electron density, its Laplacian, and the energy density. Molecular electrostatic potentials (MEPs) were calculated on the 0.001 au isodensity surface at the MP2/aug-cc-pVDZ level using the WFA-SAS program (Bulat et al., 2010).

## Results and Discussion

### Structures and MEPs of Monomers

**Figure 1:**
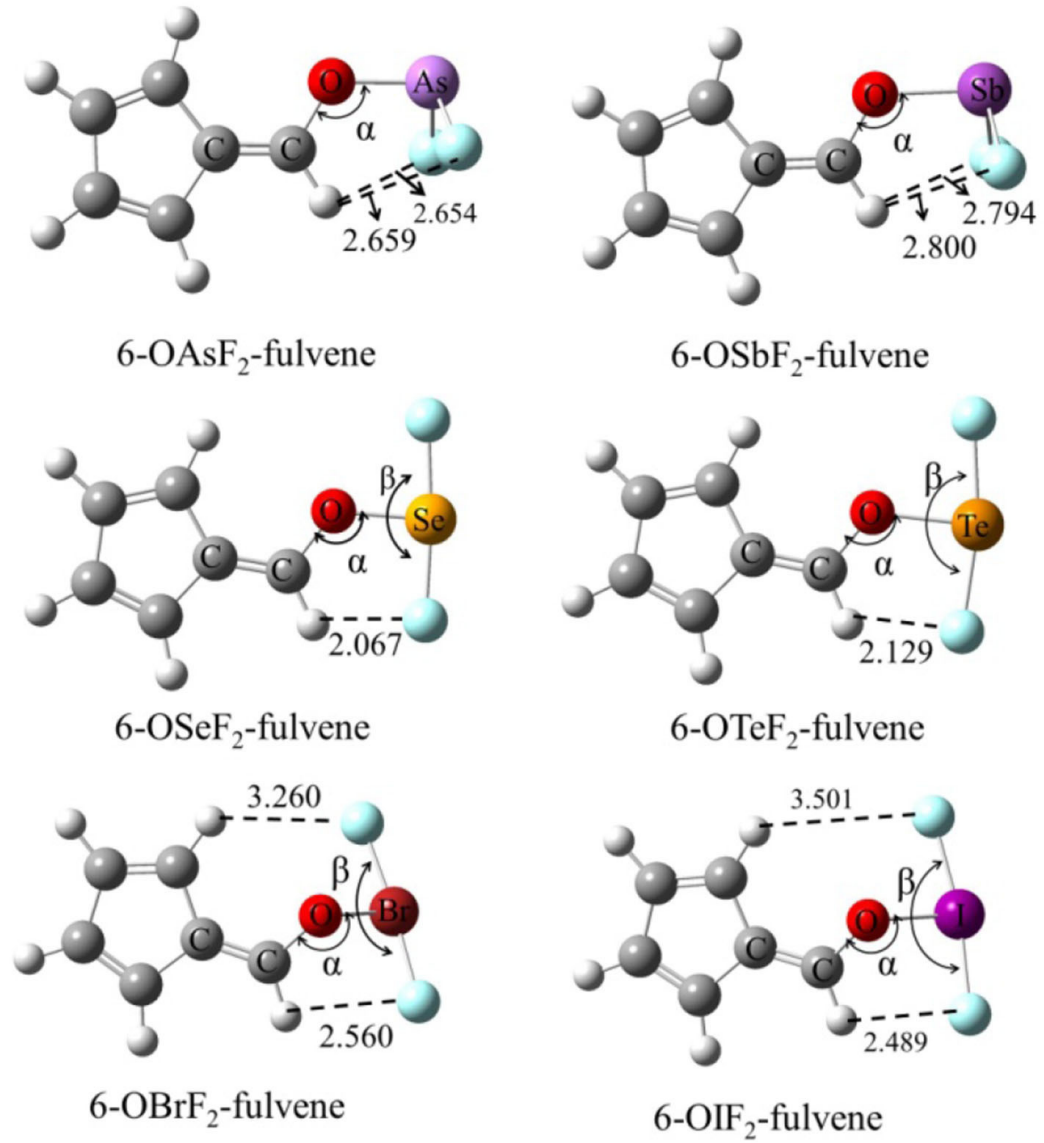
The structures of monomers and distances in Å.

Figure 1 shows the structures of six Lewis acid molecules. This figure shows that the structures of the Lewis acid molecules formed by different X atoms are not the same. For the As and Sb monomers, the two F atoms are oriented toward (and are about the same distance away from) the H atom of the -C=C-H group. This indicates that the molecular structures of the As and Sb monomers possess Cs symmetry. The F atoms of both molecules are negatively charged, so they attract the H atoms. The F···H distance is shorter in the As monomer, which suggests that the attraction is stronger in this molecule. The Cs symmetry can also be measured by the C=C-O-X dihedral angle (Table 1). For As and Sb monomers, this dihedral angle is 180°, indicating that the X atom is coplanar with the plane of the fulvene ring.

**Table 1:** Angles of C-O-X (α) and F-X-F (β) as well as C=C-O-X dihedral angle (θ) in the monomers, all in deg.

**Monomers**	**α**	**β**	**θ**
6-OAsF_2_-fulvene	120.2	93.5	179.9
6-OSbF_2_-fulvene	121.2	92.0	179.9
6-OSeF_2_-fulvene	122.3	175.2	177.2
6-OTeF_2_-fulvene	123.2	169.4	180.0
6-OBrF_2_-fulvene	112.8	175.2	127.2
6-OIF_2_-fulvene	123.2	170.5	132.8

For the Se and Te monomers, one of the F atoms is also close to the H atom of the -C=C-H group, and its separation (~2.1 Å) is much shorter than the corresponding separation in the As and Sb monomers. This value is smaller than the sum of the van der Waals (vdW) radius of the corresponding atoms (2.45 Å), suggesting that the F···H interaction is not weak. This F···H interaction makes the F-X-F arrangement non-linear, and the corresponding F-X-F angle is smaller in the Te monomer. The C=C-O-X dihedral angle shows that the Te atom is coplanar with the fulvene skeleton, while the Se atom deviates slightly from this plane.

For the Br and I monomers, the halogen atom is completely turned away from the plane of the fulvene skeleton since the C=C-O-X dihedral angle is about 130°. The Br atom deviates more than the I atom, with a smaller C=C-O-X dihedral angle for the former. Similarly, the F-X-F group is not arranged in a straight line and the F-I-F arrangement is even more non-linear. The F···H interaction also exists in these two molecules, but it is weaker than it is in the Se and Te monomers.

For 6-OXF_2_-fulvene, the C-O-X angle increases slightly when X goes from a Group V to VI to VII atom, but it decreases by about 10° only in the Br monomer. The C=C-O-X dihedral angle is significantly reduced in the molecule with the Group VII atom, especially in the Br monomer. The F···H interaction is strongest for the Group VI atom and weakest for the Group V atom.

**Figure 2:**
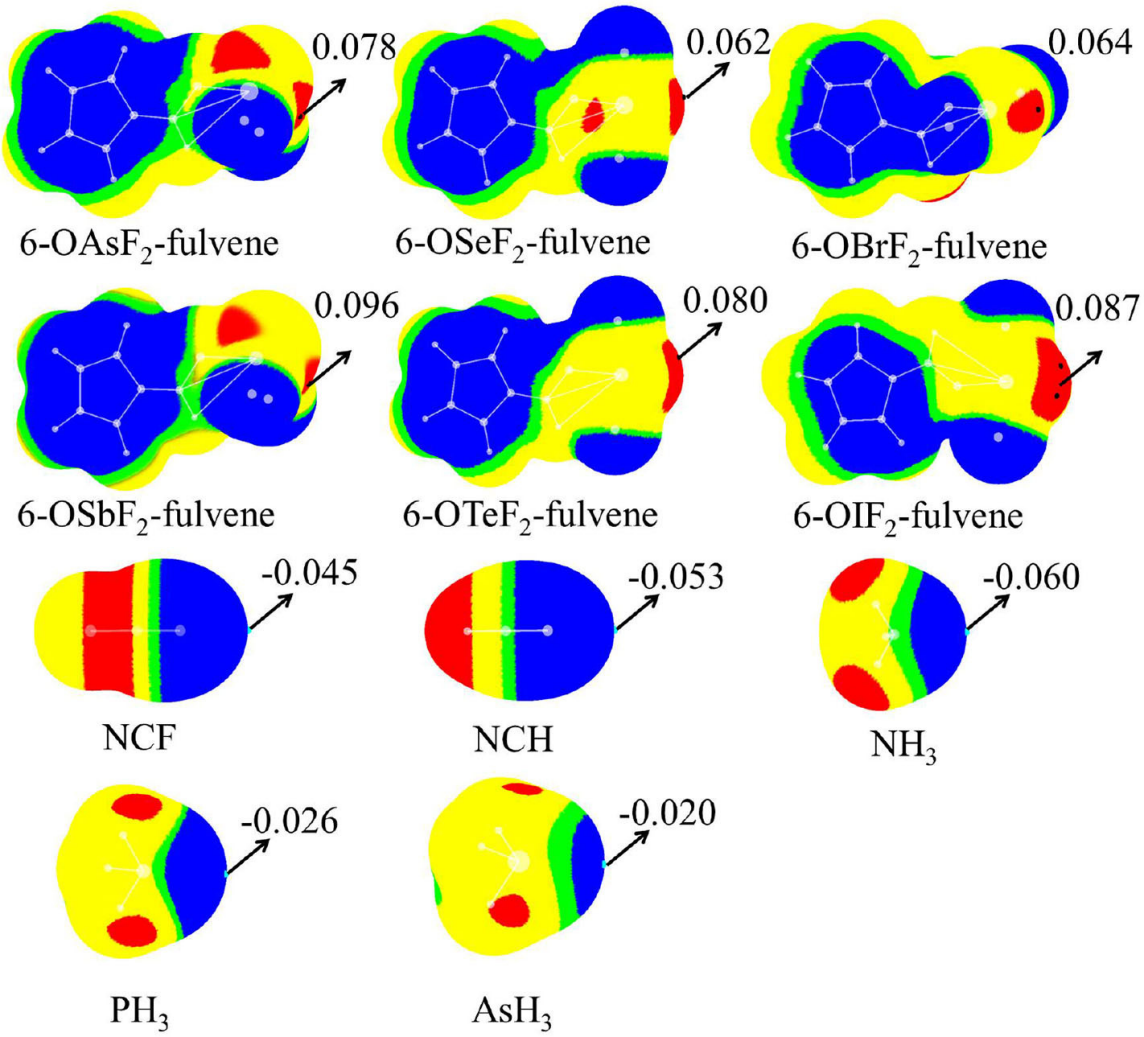
MEP maps of monomers. Color ranges are: red, larger than 0.02; yellow, between 0.02 and 0; green, between 0 and −0.002; blue, <-0.002. All are in a.u.

Figure 2 shows the MEP diagrams of all monomers with their MEP values. In 6-OXF_2_-fulvene, there is a positive MEP region (σ-hole) at the O-X bond end, and the F atom has a negative MEP due to its large electronegativity. For the same group, the σ-hole increases as the atom size increases (going down the group). For the same period, the σ-hole first decreases, then increases, with the maximum appearing for the Group V atom. Atomic radius decreases and electronegativity increases going from left to right along a particular period. A Group VI atom is difficult to polarize and thus has a smaller σ-hole. The F···H interaction for the Group VI and VII atoms reduces the F electron-withdrawing ability and thus enlarges the σ-hole. The Group VII atom has a weaker F···H interaction and thus a smaller σ-hole.

The last two rows in Figure 2 are the MEP diagrams of the Lewis base molecules. For the nitrogen-containing bases, HCN and NH_3_ are often used as electron donors in studying non-covalent interactions, and HCN is less basic than NH_3_. To further reduce its electron-donating ability, the H atom of HCN is replaced by an F atom. The negative MEP on the N atom confirms their relative basicities. For different types of ZH_3_ Lewis bases, as Z increases, the negative MEP gradually decreases, consistent with the electronegativity of Z.

### Geometries of Complexes

**Figure 3:**
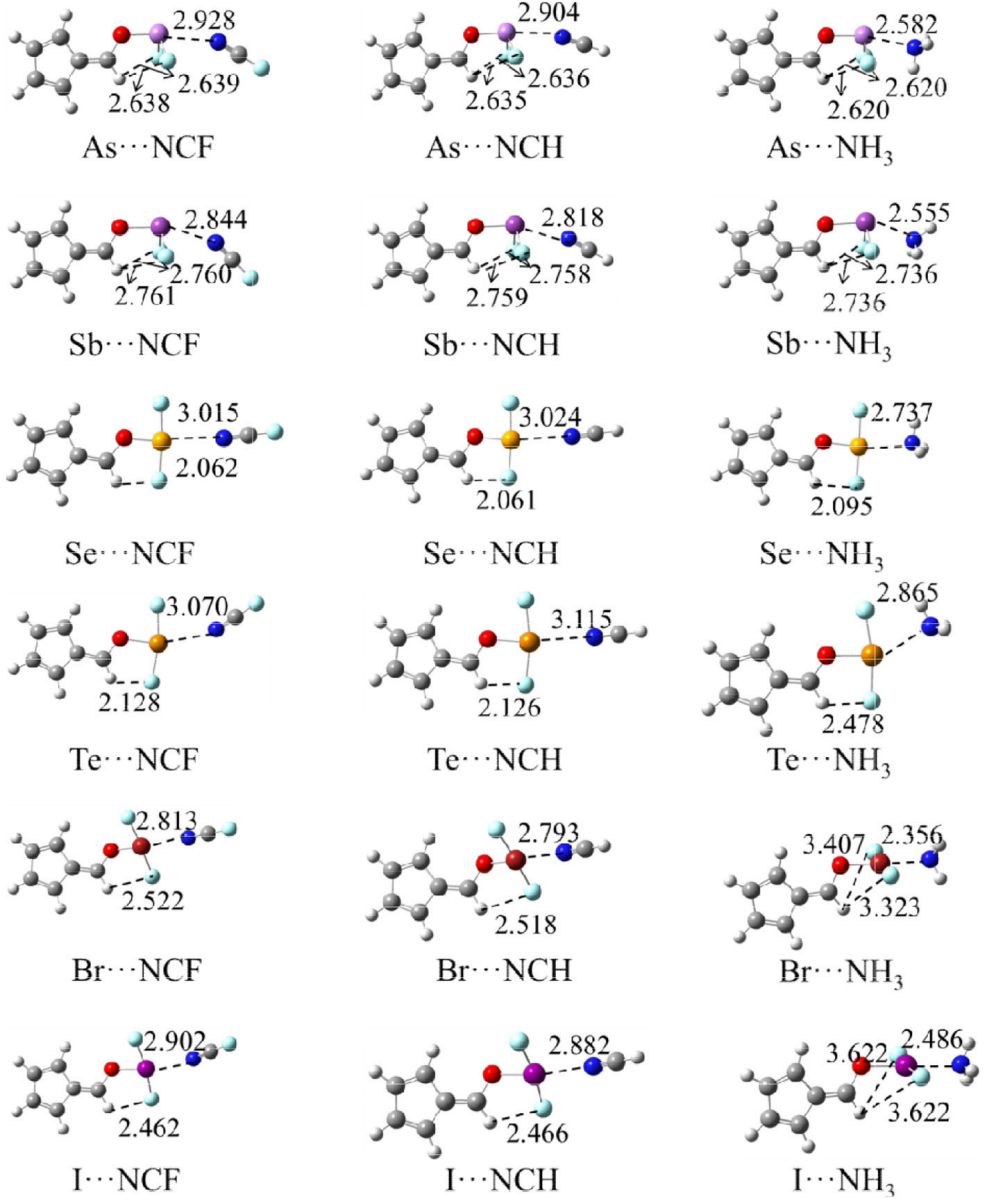
The optimized structures of complexes, distances in Å.

Figure 3 shows the structures of the complexes. We first focus on the linear arrangement involving the N and X atoms, which can be measured by the O-X···N angle in Table 2. This angle ranges between 154 and 178°, with a larger angle indicating a more linear arrangement. For the same type of interaction with a heavier X atom, the O-X···N angle decreases in almost all complexes. The O-X···N angle in the NH_3_ complex is not the largest, contrary to our expectations: i.e., the stronger the interaction, the more linear the arrangement. The linear HCN and FCN molecules are obviously not on the same line as the O-X bond. For the ZB complex, HCN and FCN are generally biased toward the two F atoms of 6-OXF_2_-fulvene. For the ChB complex, HCN and FCN are generally in the same plane as the fulvene ring and biased to one F atom of 6-OXF_2_-fulvene, particularly for FCN. For the XB complex, neither HCN nor FCN is in the same plane as the fulvene ring because the O-X bond has deviated from the plane of the fulvene ring. In the two ZB complexes with NH_3_, the configurations involving the F atoms of -AsF_2_/-SbF_2_ and the H atoms of NH_3_ are different; in the former a crossed arrangement is evident, while in the latter an overlapping arrangement is evident. For the ChB and XB complexes with NH_3_, one N-H bond is coplanar with one of the X-F bonds.

**Table 2:** Angles of C-O-X (α), F-X-F (β), and O-X···N (γ), C=C-O-X dihedral angle (θ) in the complexes as well as their difference relative to the monomers (Δ), and ratio (χ) of the X···N distance relative to the sum of vdW radii of both atoms, all in deg.

**Complexes**	**α**	**Δα**	**β**	**Δβ**	**γ**	**θ**	**Δθ**	**χ**
As-NCF	120.5	0.3	93.5	0	171.2	180.0	0.1	0.86
Sb-NCF	121.6	0.4	92.0	0	163.5	180.0	0.1	0.82
Se-NCF	122.2	−0.1	175.1	−0.1	171.9	179.9	2.7	0.87
Te-NCF	123.1	−0.1	169.0	−0.4	158.6	178.7	−1.3	0.85
Br-NCF	112.6	−0.2	175.4	0.2	177.4	137.8	10.6	0.83
I-NCF	115.0	−8.2	170.9	0.4	168.8	145.1	12.3	0.82
As-NCH	120.5	0.3	93.7	0.2	171.4	180.0	0.1	0.85
Sb-NCH	121.7	0.5	92.4	0.4	163.7	179.9	0	0.82
Se-NCH	122.2	−0.1	175.0	−0.2	172.0	179.9	2.7	0.88
Te-NCH	122.9	−0.3	169.3	−0.1	171.3	179.6	−0.4	0.86
Br-NCH	112.6	−0.2	175.6	0.4	175.2	139.8	12.6	0.82
I-NCH	115.1	−8.1	171.3	0.8	175.6	147.3	14.5	0.82
As-NH_3_	121.2	1.0	94.3	0.8	166.4	180.0	0.1	0.76
Sb-NH_3_	122.6	1.4	93.8	1.8	157.0	180.0	0.1	0.74
Se-NH_3_	121.4	−0.9	168.9	−6.3	164.9	174.2	−0.3	0.79
Te-NH_3_	122.4	−0.8	170.3	0.9	154.2	179.9	−0.1	0.79
Br-NH_3_	113.3	0.5	173.4	−1.8	176.0	176.1	48.9	0.69
I-NH_3_	116.0	−7.2	169.9	−0.1	177.2	179.7	46.9	0.70

When the X atom is fixed, the X···N distance shortens for the stronger nitrogen electron donor in most cases. However, for the ChB complex, the binding distance increases from FCN to HCN. For all interactions, when the nitrogen base goes from FCN to HCN, the binding distance hardly changes, rarely exceeding 0.03 Å; in going from HCN to NH_3_, the binding distance changes greatly, by up to 0.4 Å. The heavier X atom generally results in a longer X···N distance, however, for ZB, the Sb···N distance is shorter than the As···N distance.

Complex formation affects the structure of 6-OXF_2_-fulvene. For most complexes formed by the weak bases HCN and FCN, the C-O-X angle is almost unchanged, and it is reduced by ~8° only in the I-NCF and I-NCH complexes where the O-I bond deviates from the fulvene ring. The change of the C-O-X angle can be observed in the NH_3_ complex although its change is not consistent. Similarly, the largest reduction of the C-O-X angle is found in I-NH_3_.

In most complexes, the F-X-F angle hardly changes, especially for the weak bases HCN and FCN. Only in some complexes formed by NH_3_, this angle has an observed change, such as a reduction of 6° in Se-NH_3_. The configuration of the -AsF_2_ and -SbF_2_ groups in the complex are almost the same as in the monomer since the C-O-X and F-X-F angles, as well as the C=C-O-X dihedral angle, show little change. In the ZB complex, the F···H distance is shortened and its shortening is larger for the stronger Lewis base. In the ChB complex, the relative configuration of the -XF_2_ group is similar to that in the monomer since the change of the C=C-O-X dihedral angle, as well as the C-O-X and F-X-F angles, is small. In most ChB complexes, the F···H distance is also shortened, but it is increased in the NH_3_ complexes. Although the F-X-F angle varies little in the XB complex, it has a large change with respect to the fulvene skeleton since the change in the C=C-O-X dihedral angle is substantial. This dihedral angle has a larger change for the stronger nitrogen base. In the Br···NH_3_ and I···NH_3_ complexes, the F-X-F group is perpendicular to the plane containing the fulvene ring. Similarly, the F···H distance in most XB complexes has a similar change to that in the ChB complexes.

### Interaction Energies of Complexes

Table 3 presents the interaction energy (E_int_), binding energy (E_b_), and deformation energy (DE) for all complexes. The binding energy is the difference in the energy of the complex relative to the sum of the energies for the isolated monomers (in their optimized geometries). In general, the trend for E_b_ is the same as for E_int_, but E_b_ is smaller than E_int_, and their difference is DE. The FCN and HCN complexes have a small DE, not exceeding 0.5 kcal/mol due to the weak interaction. The NH_3_ complex shows a large DE, especially for XB where DE is equivalent to 30% of E_int_, consistent with the structural change described above. Such a large contribution of DE to E_int_ is seldom found for XBs with conventional halogen donors.

**Table 3:** Interaction energy (E_int_), binding energy (E_b_), and deformation energy (DE), all in kcal/mol.

**Complexes**	**E_**int**_**	**E_**b**_**	**DE**
As-NCF	−3.87	−3.68	0.19
Sb-NCF	−6.00	−5.60	0.40
Se-NCF	−3.66	−3.58	0.07
Te-NCF	−4.78	−4.66	0.12
Br-NCF	−4.99	−4.81	0.18
I-NCF	−6.29	−6.05	0.24
As-NCH	−4.30	−4.06	0.24
Sb-NCH	−6.60	−6.14	0.46
Se-NCH	−4.05	−3.94	0.11
Te-NCH	−5.29	−5.13	0.16
Br-NCH	−5.61	−5.41	0.20
I-NCH	−7.09	−6.83	0.26
As-NH_3_	−8.51	−7.22	1.29
Sb-NH_3_	−14.26	−12.28	1.99
Se-NH_3_	−7.19	−6.42	0.77
Te-NH_3_	−8.71	−8.28	0.42
Br-NH_3_	−15.97	−10.62	5.34
I-NH_3_	−18.00	−12.50	5.50

**Figure 4:**
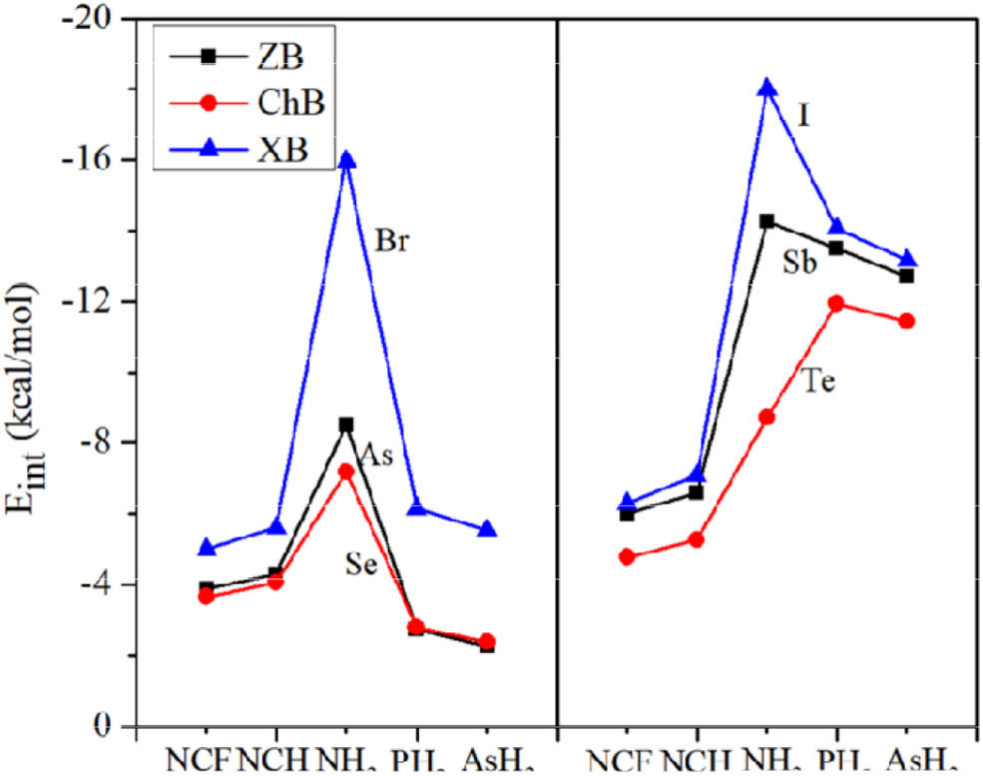
Plots of the interaction energy (E_int_) for different bases.

Figure 4 plots the variation of the interaction energy for different systems. For each type of interaction, E_int_ gradually increases for the stronger nitrogen base, which is consistent with the negative MEP on the N atom. From FCN to HCN, the increase of E_int_ is small (not exceeding 1 kcal/mol), while from HCN to NH_3_, the increase of E_int_ is large, up to 11 kcal/mol. The E_int_ of the NH_3_ complex is increased by 65–185% relative to the HCN analog. This percentage increase is smallest for ChB and largest for XB. For the same type of interaction, E_int_ increases with the increase of X atomic mass, consistent with the σ-hole on the X atom. Regardless of the Lewis base, the interaction becomes stronger in the order ChB < ZB < XB, with some inconsistencies regarding the magnitude of the σ-hole on the X atom, due mainly to large structural changes in the halogen-containing molecule. This shows that the strength of the non-covalent bond is determined not only by the electrostatic potential, but also by other factors.

In Figure 4, the interaction energy for the complexes of PH_3_/AsH_3_ is also plotted. The interactions are weaker for the heavier pnicogen atom, excluding Te-NH_3_, consistent with the negative MEP on the pnicogen atom. We compare the interaction strength of FCN/HCN with PH_3_/AsH_3_. The former has the more negative MEP, thus it forms a stronger ZB with As and ChB with Se. However, the opposite is found for the XB with Br. This abnormal result is also found for all three types of interactions when X = Sb, Te, and I, and is pronounced since the interaction energy with PH_3_/AsH_3_ is about twice as large as that for FCN/HCN.

In most complexes, the interaction energy is a net result of more than one interaction. To estimate the change of X···N interaction strength, the ratio of X···N distance relative to the sum of the van der Waals radii of both atoms is calculated (Table 2). In most cases, the smaller ratio corresponds to the larger interaction energy and thus a strong X···N interaction. However, some exceptions are present. For example, both Br-NCH and I-NCH have an equivalent ratio, but their interaction energies show an obvious difference.

There are some studies available comparing the strengths of ZB, ChB, and XB, which allow us to compare our results with those from previous studies. Dong and coauthors performed a detailed comparison for the XB, ChB, ZB, and tetrel bond (TB) in complexes of BrH, SeH_2_, AsH_3_, and GeH_4_ with NH_3_, respectively. The ChB is the strongest, followed by XB, ZB, and TB. The replacement of the atom opposite the base by F changes the order to XB > ChB > ZB > TB (Dong et al., 2018). Both orderings are different from our results. This indicates that the number and type of substituent adjoined to the acidic center has an important effect on the strength of the interaction.

### Origin of Interactions

**Figure 5:**
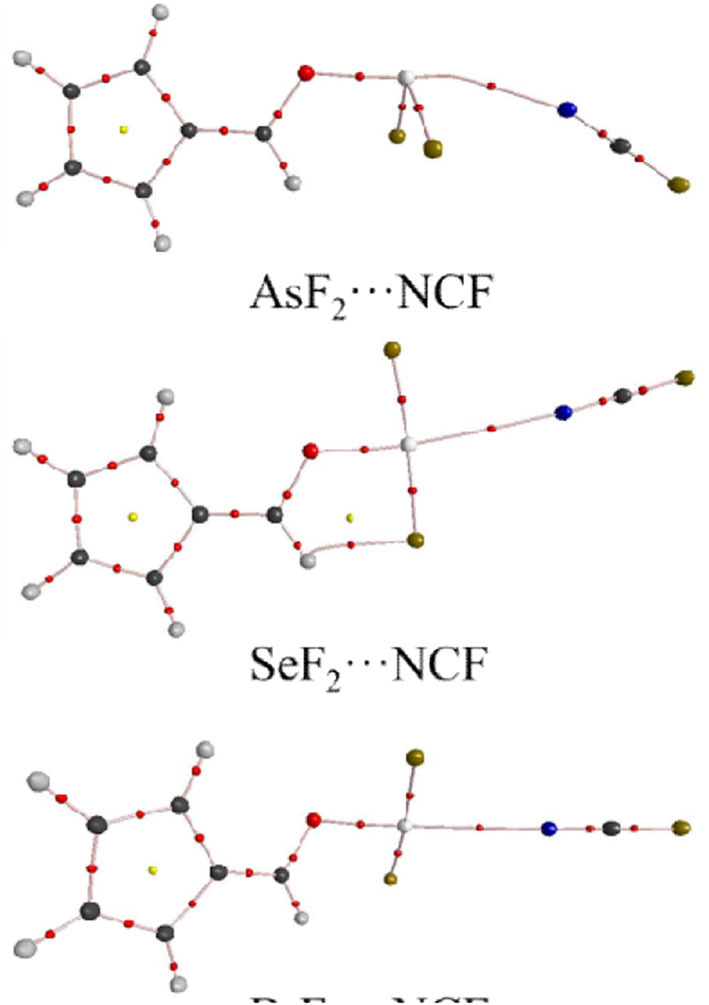
AIM diagrams for three different types of complexes.

**Figure 6:**
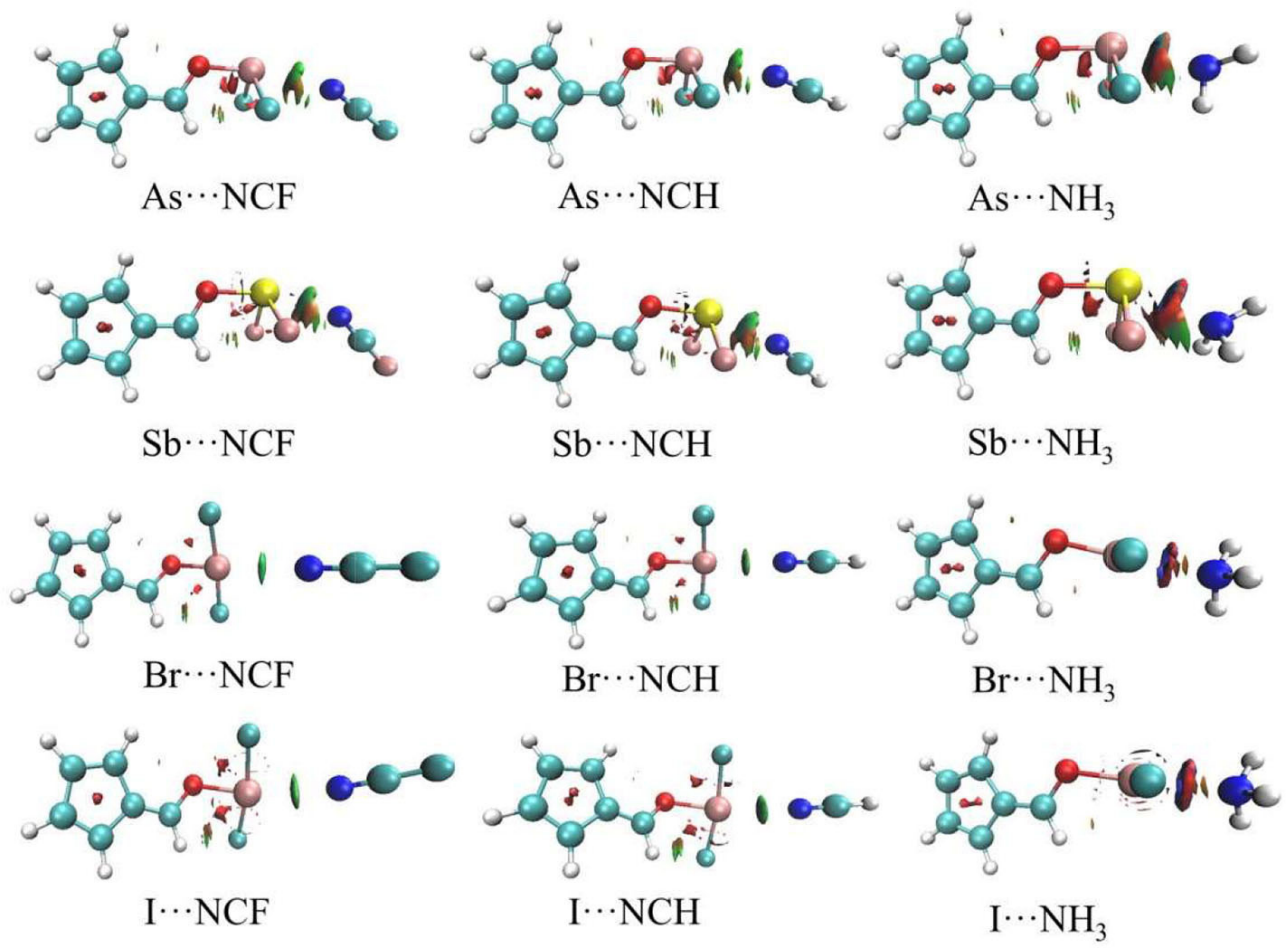
NCI diagrams for ZB and XB types of complexes.

Figure 5 shows the AIM diagrams for the three types of complexes formed by NCF; the types of complexes for the other bases are similar. For each type of interaction, an intermolecular bond critical point (BCP) is found along the X···N path, confirming the formation of the corresponding non-covalent interaction. There is also a F···H BCP in the ChB complex, indicating the existence of a F···H interaction. This BCP is not found in the ZB and XB complexes since the F···H interaction is very weak. Actually, such weak interaction can be confirmed by the green region between the F and H atoms in the NCI analyses (Figure 6).

The topological data including electron density (ρ), Laplacian (∇^2^ρ), and total energy density (H) at the X···N BCP are collected in Table 4. For the complexes with the two weaker bases NCF and NCH, both ∇^2^ρ and H are positive, which indicates that the corresponding non-covalent bond belongs to a completely closed shell interaction, consistent with its relatively weak nature. For the complexes with NH_3_, except Se-NH_3_, ∇^2^ρ is still positive but H becomes negative, which indicates that the corresponding interaction has a partially covalent character (Arnold and Oldfield, 2000). As the base goes from FCN to HCN to NH_3_, the positive value for H gradually decreases and even becomes negative, while ∇^2^ρ generally becomes larger. For the same type of interaction with a heavier X atom and a fixed base, the positive H decreases, while the negative H becomes more negative. Therefore, ∇^2^ρ not only characterizes the type of interaction (by its sign) but can also gauge the interaction strength by its magnitude.

**Table 4:** Electron density (ρ), Laplacian (Δ^2^ρ), and total energy density (H) in the complexes, all in a.u.

**Complexes**	**ρ**	**∇^2^ρ**	**H**
As-NCF	0.014	0.0416	0.0010
Sb-NCF	0.019	0.0524	0.0005
Se-NCF	0.013	0.0458	0.0017
Te-NCF	0.014	0.0445	0.0011
Br-NCF	0.018	0.0710	0.0026
I-NCF	0.019	0.0644	0.0015
As-NCH	0.015	0.0436	0.0009
Sb-NCH	0.020	0.0558	0.0002
Se-NCH	0.013	0.0452	0.0016
Te-NCH	0.014	0.0420	0.0010
Br-NCH	0.020	0.0744	0.0026
I-NCH	0.021	0.0673	0.0013
As-NH_3_	0.032	0.0672	−0.0027
Sb-NH_3_	0.039	0.0903	−0.0045
Se-NH_3_	0.027	0.0730	0.0003
Te-NH_3_	0.025	0.0603	−0.0006
Br-NH_3_	0.058	0.1329	−0.0072
I-NH_3_	0.052	0.1087	−0.0078

For the same type of interaction, the electron density is almost the same in the complexes formed by FCN and HCN. When the nitrogen base changes from HCN to NH_3_, the electron density significantly increases and the maximal increase is 0.038 a.u in Br-NH_3_. For the different types of interactions, the electron density becomes larger in the order XB > ZB> ChB, although the intermolecular BCP is different.

Table 5 presents charge transfer (CT) and second-order perturbation energy data for the three types of complexes. For the same type of interaction, the CT value for the complex formed by the two weaker bases FCN and HCN is almost the same. When the Lewis base is NH_3_, CT increases significantly and this increase varies with the type of complex. The smallest increase is found for the ChB complex, while the largest is for the XB complex. For a fixed base, CT is smallest for ChB, while the relative CT for XB and ZB, is less straightforward but even so, XB has the largest CT for NH_3_. For the same type of interaction with the heavier X atom, CT also increases, but an unusual result occurs for Br-NH_3_, which has the largest CT (0.142e). Generally, there is correlation between CT and E_int_.

**Table 5:** Charge transfer (CT, e) and second-order perturbation energy (E^2^, kcal/mol) in the complexes.

**Complexes**	**CT**	**Orbital type**	**E^**2**^**
As-NCF	0.008	Lp_N_ → BD*_O−As_	4.07
Sb-NCF	0.017	Lp_N_ → Lp*_Sb_	6.81
Se-NCF	0.003	Lp_N_ → BD*_O−Se_	1.40
Te-NCF	0.003	Lp_N_ → Lp*_Te_	1.86
Br-NCF	0.009	Lp_N_ → BD*_O−Br_	4.24
I-NCF	0.012	Lp_N_ → BD*_O−I_	4.95
As-NCH	0.008	Lp_N_ → BD*_O−As_	4.56
Sb-NCH	0.017	Lp_N_ → Lp*_Sb_	13.99
Se-NCH	0.003	Lp_N_ → BD*_O−Se_	1.37
Te-NCH	0.005	Lp_N_ → Lp*_Te_	1.73
Br-NCH	0.011	Lp_N_ → BD*_O−Br_	4.95
I-NCH	0.014	Lp_N_ → BD*_O−I_	7.22
As-NH_3_	0.061	Lp_N_ → BD*_O−As_	18.17
Sb-NH_3_	0.079	Lp_N_ → Lp*_Sb_	45.86
Se-NH_3_	0.021	Lp_N_ → BD*_O−Se_	6.02
Te-NH_3_	0.021	Lp_N_ → Lp*_Te_	6.31
Br-NH_3_	0.142	Lp_N_ → BD*_O−Br_	54.58
I-NH_3_	0.133	Lp_N_ → BD*_O−I_	53.60

For XB, the main orbital interaction is Lp_N_ → BD*_O-X_, where Lp_N_ represents the lone pair orbital on the N atom and BD*_O-X_ is the anti-bonding orbital of O-X bond. For ZB and ChB, the main orbital interaction is also Lp_N_ → BD*_O-X_ when X = As and Se, but it is changed to Lp_N_ → Lp*_X_ (Lp*_X_ the empty orbital of the X atom) when X = Sb and Te. If the base is stronger, the corresponding orbital interaction is also stronger except the ChB formed by HCN. For the same type of interaction with the heavier X atom, the perturbation energy is larger excluding Br-NH_3_. When NH_3_ acts as a base, the orbital interaction is strongest in XB and weakest in ChB. When the Lewis base is FCN and HCN, ChB also has the weakest orbital interaction, while the relative strength of orbital interaction between XB and ZB is a little complicated.

**Figure 7:**
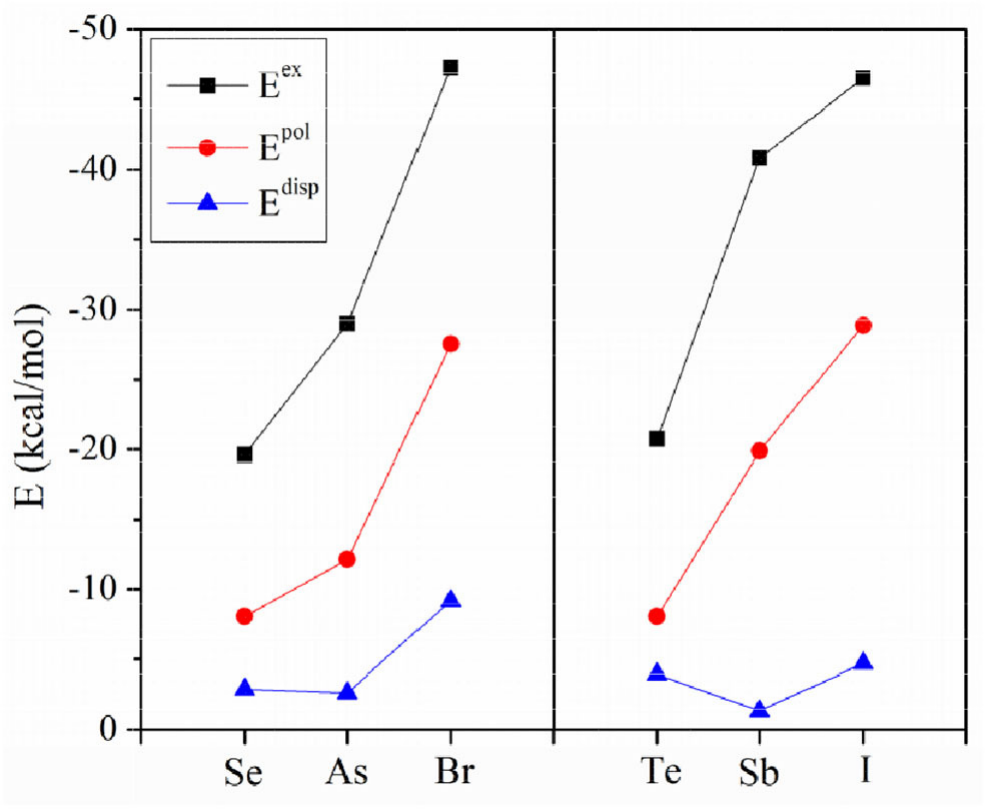
Three attractive energy components.

To assess the similarities and differences between ZB, ChB and XB, the interaction energy was decomposed into electrostatic (E^ele^), exchange (E^ex^), repulsion (E^rep^), polarization (E^pol^) and dispersion energies (E^disp^), as shown in Table 6. The three attractive terms (E^ele^, E^pol^, and E^disp^) in the NH_3_ complexes are plotted in Figure 7. The pattern is basically the same for the other two Lewis bases. Each term has a similar trend with the interaction energy. Whether XB or ZB and ChB, the electrostatic energy is dominant, indicating that the three types of interactions are electrostatic in nature. When the base is FCN or HCN, both E^pol^ and E^disp^ are comparable in magnitude. When the base is NH_3_, E^pol^ is larger than E^disp^. This indicates that the increase in the interaction energy is partly due to the increase in the polarization energy. Each term becomes more negative going from ChB to ZB to XB. For some complexes, the interaction energy is not consistent with the σ-hole MEP on the X atom, but the electrostatic interaction is still dominant in all complexes. This inconsistency is mainly attributable to the structural change of 6-OXF_2_-fulvene in the complex relative to the monomer.

**Table 6:** Electrostatic (E^ele^), exchange (E^ex^), repulsion (E^rep^), polarization (E^pol^), and dispersion energies (E^disp^) in the complexes, all in kcal/mol.

**Complexes**	**E^**ele**^**	**E^**ex**^**	**E^**rep**^**	**E^**pol**^**	**E^**disp**^**
As-NCF	−7.02	−10.06	17.91	−2.38	−2.32
Sb-NCF	−11.84	−17.00	30.87	−5.27	−2.35
Se-NCF	−5.91	−7.63	13.62	−1.83	−1.91
Te-NCF	−7.75	−10.20	18.13	−2.65	−2.88
Br-NCF	−8.03	−11.47	21.11	−3.14	−3.49
I-NCF	−10.41	−13.85	25.32	−4.42	−2.95
As-NCH	−8.06	−11.23	20.06	−2.80	−2.27
Sb-NCH	−13.48	−18.83	34.35	−6.03	−2.27
Se-NCH	−6.48	−7.81	13.92	−2.00	−1.69
Te-NCH	−8.14	−9.70	17.21	−2.67	−2.63
Br-NCH	−9.17	−12.77	23.60	−3.68	−3.62
I-NCH	−11.94	−15.42	28.31	−5.14	−2.93
As-NH_3_	−29.01	−39.45	74.74	−12.15	−2.63
Sb-NH_3_	−40.83	−53.26	101.74	−19.87	−1.33
Se-NH_3_	−19.64	−26.84	50.21	−8.06	−2.87
Te-NH_3_	−20.74	−27.76	50.78	−8.05	−3.93
Br-NH_3_	−47.25	−66.03	133.91	−27.53	−9.18
I-NH_3_	−46.51	−63.43	125.49	−28.88	−4.71

## Conclusions

Three different types of complexes formed between 6-OXF_2_-fulvene (X = As, Sb, Se, Te, Br, and I) and the three N bases FCN, HCN and NH_3_ were studied. The three types of interactions operative in the complexes have a predominantly electrostatic nature with differing strengths. The relative ordering for these interactions is halogen bond > pnicogen bond > chalcogen bond. Each type of interaction becomes stronger when the N base varies from FCN to HCN to NH_3_, consistent with the negative MEP on the N atom. The strength of each type of interaction is also consistent with electron density, charge transfer and orbital interaction analyses. The heavier X atom engages in a stronger interaction than its lighter analog, and the relative degree of enhancement depends on the type of interaction. For the stronger interactions, the polarization contribution becomes more dominant, as confirmed by the negative energy density at the corresponding intermolecular BCP, and this relatively large polarization causes a structural change of 6-OXF_2_-fulvene, particularly for X = halogen.

## Data Availability Statement

The raw data supporting the conclusions of this article will be made available by the authors, without undue reservation.

## Author Contributions

QL: conceptualization, software, resources, data curation, supervision, project administration, and funding acquisition. NL: methodology, formal analysis, investigation, writing—original draft preparation, and visualization. NL and QL: validation. QL and SM: writing—review and editing. All authors: contributed to the article and approved the submitted version.

## Conflict of Interest

The authors declare that the research was conducted in the absence of any commercial or financial relationships that could be construed as a potential conflict of interest.
