# Supplementary material for: Reliable Comparison of Pnicogen, Chalcogen, and Halogen Bonds in Complexes of 6-OXF2-Fulvene (X = As, Sb, Se, Te, Be, I) With Three Electron Donors
Source: Front Chem. 2020 Dec 9;8:608486. doi: 10.3389/fchem.2020.608486 (PMC7793776; doi:10.3389/fchem.2020.608486)
Supplement: Supplementary file 1 [file Table_1.DOCX]

NF

C 0.00000000 0.00000000 -0.14784600

N 0.00000000 0.00000000 -1.33480600

F 0.00000000 0.00000000 1.13674600

NH

C 0.00000000 0.00000000 -0.51438300

N 0.00000000 0.00000000 0.66842700

H 0.00000000 0.00000000 -1.59268800

NH_3_

N 0.00000000 0.00000000 0.11702000

H 0.00000000 0.94290200 -0.27304600

H -0.81657700 -0.47145100 -0.27304600

H 0.81657700 -0.47145100 -0.27304600

PH3

P 0.00000100 0.00009100 -0.12812900

H -1.04308800 0.59707600 0.64093100

H 1.03918300 0.60382500 0.64094200

H 0.00389400 -1.20226400 0.64006500

AsH3

H 1.09864600 -0.63743300 0.76238900

H -1.10136700 -0.63272100 0.76238900

H 0.00272000 1.27017900 0.76237300

As 0.00000000 -0.00000100 -0.06930800

As-NF

C -3.82386600 -0.86078600 -0.00008800

C -5.12038900 -0.38336900 -0.00018400

C -5.09615200 1.08516700 -0.00006300

C -3.78299600 1.50876600 0.00013300

H -3.49763600 -1.90094200 -0.00011900

H -6.02620000 -0.99427400 -0.00032900

H -5.97886400 1.72824200 -0.00013400

H -3.41504000 2.53628800 0.00022000

C -2.94151600 0.30539900 0.00012200

C -1.57710600 0.33264300 0.00020900

H -1.01342200 1.27172400 0.00035500

O -0.85041700 -0.82924400 0.00010100

As 0.95807000 -0.78201000 -0.00009700

F 1.12557500 0.41251100 -1.28256400

F 1.12589800 0.41170800 1.28307600

C 4.82769100 0.39512100 -0.00018200

N 3.83827000 -0.25659900 -0.00046200

F 5.89700500 1.09775800 0.00015100

Sb-NF

C -4.03829200 -0.72195900 -0.00086400

C -5.31764000 -0.19572800 -0.00113800

C -5.23758400 1.26868800 -0.00018000

C -3.90722300 1.64057000 0.00068700

H -3.75212600 -1.77394800 -0.00139600

H -6.24592100 -0.77220600 -0.00194500

H -6.09462800 1.94575800 -0.00017900

H -3.49965400 2.65329800 0.00148700

C -3.11264300 0.40779500 0.00028300

C -1.74470800 0.38368800 0.00086600

H -1.16140200 1.31322500 0.00169200

O -1.05401500 -0.78673400 0.00048200

Sb 0.94206500 -0.82446900 0.00006400

F 1.08529300 0.50787200 1.38864600

F 1.08467100 0.50881400 -1.38767100

C 4.52033600 0.76880000 -0.00061300

N 3.68413700 -0.06842600 -0.00060400

F 5.42760500 1.66592900 -0.00062000

Se-NF

C 3.63513100 1.11447600 0.00139400

C 5.00833300 0.97940900 0.00275200

C 5.35573800 -0.45308900 0.00284800

C 4.19193200 -1.19397500 0.00155800

H 3.05541100 2.03759000 0.00104200

H 5.73172500 1.79787900 0.00366100

H 6.37213400 -0.85245000 0.00375900

H 4.09254300 -2.28044900 0.00120500

C 3.07668100 -0.23880900 0.00061900

C 1.75947200 -0.61314900 -0.00072100

H 1.40443400 -1.64396500 -0.00120600

O 0.80842100 0.39347300 -0.00161700

Se -0.96261700 0.04374000 -0.00216000

F -0.65129100 -1.80414900 -0.00205600

F -1.11033700 1.87041800 -0.00202200

C -5.15826500 -0.08817300 0.00256100

N -3.97351700 -0.11672400 0.00002700

F -6.43772100 -0.05700400 0.00537600

Te-NF

C 3.69736900 -1.38118200 0.00092100

C 5.07788100 -1.41087400 0.02528800

C 5.59218300 -0.03240100 0.05115200

C 4.52366300 0.84153200 0.04230100

H 3.01193400 -2.22867800 -0.02188100

H 5.69843700 -2.31006400 0.02544900

H 6.64866000 0.24369100 0.07404200

H 4.55437700 1.93233900 0.05666600

C 3.30287000 0.02731800 0.01075600

C 2.03781200 0.55250300 -0.00443200

H 1.82602400 1.62443600 0.00513400

O 0.96894100 -0.31031800 -0.03540700

F -0.22505900 2.19246500 -0.00480400

F -1.21903900 -1.65619400 -0.05581600

C -5.06920700 -0.34007800 0.04817800

N -3.97532200 0.11411700 0.03124900

F -6.24884000 -0.83095300 0.06639800

Te -0.91159000 0.29871600 -0.02253600

Br-NF

C 3.41254200 -0.99551200 -0.37437100

C 4.70049600 -1.12788700 0.09717000

C 5.01691600 0.01076000 0.97869700

C 3.91958200 0.83938200 1.05201200

H 2.86477100 -1.67196500 -1.03008400

H 5.38342500 -1.94909500 -0.13136700

H 5.96926900 0.16100900 1.49164400

H 3.82060700 1.76808400 1.61581200

C 2.87436800 0.23816900 0.20911800

C 1.64532100 0.78931600 0.00573100

H 1.28582700 1.70729500 0.48298100

O 0.81998400 0.23857500 -0.95701100

Br -0.92099000 0.11106300 -0.40263700

F -0.68019100 -1.73796200 -0.29229700

F -1.01374700 1.98956100 -0.51316000

N -3.62340700 -0.13999200 0.33541500

C -4.76051900 -0.28249300 0.63404400

F -5.98801000 -0.43622200 0.95624400

I-NF

C 3.62723000 -1.14283700 -0.43497000

C 4.92701900 -1.33381900 -0.01291300

C 5.30325800 -0.25080900 0.90956500

C 4.23120000 0.60302200 1.05713100

H 3.04301400 -1.76972200 -1.10857000

H 5.57888000 -2.16008800 -0.30567800

H 6.27541700 -0.15045900 1.39697900

H 4.17665100 1.50621400 1.66702300

C 3.14411600 0.07386800 0.22108700

C 1.92381800 0.66954700 0.09324300

H 1.63559700 1.58257500 0.62638700

O 1.02759400 0.18482600 -0.83412800

I -0.88414900 0.28367600 -0.28642900

F -0.58779100 2.27197100 -0.17482400

F -0.86517900 -1.70481100 -0.43933100

C -4.77199500 -0.51294900 0.61119700

N -3.68262800 -0.11989300 0.36695500

F -5.94702000 -0.93592300 0.87447500

As-NH

C 3.35027600 -1.01056500 -0.00318900

C 4.67756500 -0.62602900 -0.00354200

C 4.75712700 0.84023000 -0.00069900

C 3.47687300 1.35543400 0.00145200

H 2.95125400 -2.02505200 -0.00488800

H 5.53793400 -1.29947000 -0.00563100

H 5.68302500 1.41944700 -0.00034400

H 3.18235400 2.40639200 0.00382000

C 2.55259000 0.21480500 -0.00003800

C 1.19307300 0.33827800 0.00139800

H 0.69849400 1.31563900 0.00379400

O 0.38560200 -0.76723500 0.00004700

As -1.41791400 -0.58946800 0.00101000

F -1.49189300 0.60999700 1.28692500

F -1.49278600 0.61362200 -1.28153700

C -5.27843300 0.67269400 -0.00679000

H -6.23221400 1.17475900 -0.01307400

N -4.23240200 0.12528200 0.00036700

Sb-NH

C -3.64655000 -0.94584900 -0.00078800

C -4.96929800 -0.53980300 -0.00117900

C -5.02445900 0.92536100 -0.00037100

C -3.73360400 1.41807100 0.00053100

H -3.26454800 -1.96698400 -0.00118100

H -5.84049400 -1.19945400 -0.00196900

H -5.94017300 1.52075300 -0.00046800

H -3.42095400 2.46404800 0.00127200

C -2.82899700 0.26406300 0.00027800

C -1.46407800 0.36594700 0.00099500

H -0.97033300 1.34616200 0.00181900

O -0.66799200 -0.73340600 0.00090700

Sb 1.32686900 -0.58532500 0.00018000

F 1.33545900 0.74961900 1.39265700

F 1.33437100 0.74969800 -1.39221500

C 4.92236200 1.09417200 -0.00132000

H 5.79568100 1.72652500 -0.00152700

N 3.96481700 0.40602600 -0.00103400

Se-NH

C -3.18439000 1.12045000 0.00033100

C -4.55796100 0.98845300 0.00089600

C -4.90845800 -0.44312300 0.00079600

C -3.74612900 -1.18649100 0.00016800

H -2.60255500 2.04224300 0.00022600

H -5.27957200 1.80851900 0.00134800

H -5.92572000 -0.84029800 0.00116000

H -3.64899300 -2.27318800 -0.00006300

C -2.62893100 -0.23385700 -0.00013000

C -1.31232000 -0.61104000 -0.00075000

H -0.96024400 -1.64293700 -0.00108500

O -0.35869800 0.39231800 -0.00120000

Se 1.41263000 0.03661300 0.00028900

F 1.09403800 -1.81074000 -0.00056100

F 1.56467900 1.86320700 -0.00101600

C 5.60846800 -0.11546900 0.00338200

H 6.68588000 -0.09346200 0.00980600

N 4.43160400 -0.13857300 -0.00365800

Te-NH

C -3.49650500 1.12818000 0.00514100

C -4.87006300 0.98266800 0.00794300

C -5.20590800 -0.44965800 0.01109300

C -4.03513300 -1.18107700 0.00925700

H -2.92393300 2.05588100 0.00281400

H -5.59933800 1.79605800 0.00830200

H -6.21902600 -0.85752100 0.01377000

H -3.92732700 -2.26702500 0.01021400

C -2.92685200 -0.21879600 0.00586500

C -1.60630600 -0.58275700 0.00426200

H -1.26557800 -1.62099900 0.00449800

O -0.64943100 0.40285500 0.00297200

Te 1.29126200 0.01785000 -0.01072800

F 1.37411000 1.99627600 -0.00520600

F 0.83511900 -1.94532000 -0.01155400

C 5.57848400 -0.13166400 0.03962300

H 6.65636900 -0.13788600 0.07007800

N 4.40275500 -0.12278600 0.01087300

Br-NH

C 3.02581200 -0.97116600 -0.39296400

C 4.34545200 -1.00505700 0.00313300

C 4.66215300 0.22508900 0.75066700

C 3.53372500 1.01181800 0.81757900

H 2.47109400 -1.72969900 -0.94483700

H 5.05043200 -1.81627400 -0.19177000

H 5.63622700 0.46284200 1.18349300

H 3.42642500 1.98599700 1.29684000

C 2.46784700 0.29021800 0.10552700

C 1.20445800 0.76993000 -0.06726800

H 0.83563700 1.71595500 0.34352900

O 0.34576900 0.09597100 -0.91452000

Br -1.36041800 -0.00511500 -0.25429200

F -1.06520100 -1.82559400 0.04625200

F -1.50894300 1.84828100 -0.56574400

N -3.99927400 -0.19349700 0.64214400

C -5.11779100 -0.27272400 1.01045100

H -6.13908400 -0.34590800 1.34679700

I-NH

C 3.36316700 -1.04730100 -0.37026400

C 4.69402800 -1.05942600 -0.00467700

C 5.01213400 0.18344500 0.71500000

C 3.87366200 0.95697100 0.79507400

H 2.80838800 -1.81991700 -0.90258100

H 5.40465300 -1.86413900 -0.20680800

H 5.99297100 0.44112600 1.12036000

H 3.76494700 1.93777800 1.26089700

C 2.80161300 0.21290800 0.11900100

C 1.52678300 0.67746200 -0.02245900

H 1.18536500 1.63797100 0.37980400

O 0.63244300 -0.02259500 -0.80043700

I -1.26298200 0.06642300 -0.19344900

F -1.09355000 2.05721000 -0.45500100

F -1.12645300 -1.91271800 0.01645700

C -5.11845200 -0.41314800 0.97925100

H -6.12926200 -0.60583300 1.30134000

N -4.01230400 -0.20465200 0.62637300

As-NH_3_

C 3.07245300 -1.07647900 -0.00064900

C 4.41134800 -0.73040500 -0.00057300

C 4.53273600 0.73170800 0.00012000

C 3.26640800 1.28282000 0.00035200

H 2.64443600 -2.07917200 -0.00106300

H 5.25197600 -1.42847800 -0.00090100

H 5.47465300 1.28465800 0.00034200

H 3.00216800 2.34196700 0.00077800

C 2.31038100 0.17006500 -0.00005700

C 0.95290200 0.33256800 0.00013100

H 0.49560400 1.32847300 0.00063600

O 0.10943300 -0.73652200 -0.00026300

As -1.70916100 -0.51155900 0.00000800

F -1.68906100 0.69032800 1.29834000

F -1.68917700 0.69254000 -1.29630000

N -4.12511600 0.40064400 -0.00105700

H -4.69180600 0.21256900 -0.82900300

H -3.94278100 1.40534700 0.01024700

H -4.70482300 0.19629100 0.81390100

Sb-NH_3_

C -3.40652500 -1.04903600 0.00001800

C -4.74619300 -0.69878700 -0.00000400

C -4.86230000 0.76156000 -0.00000900

C -3.59178400 1.30722600 0.00000100

H -2.98214200 -2.05339500 0.00002200

H -5.58903000 -1.39444800 -0.00001300

H -5.80189400 1.31867400 -0.00002100

H -3.32311600 2.36552000 0.00000500

C -2.64040400 0.19284300 0.00001300

C -1.27884900 0.35193300 0.00002400

H -0.83480300 1.35618300 0.00002400

O -0.43326000 -0.70099000 0.00004500

Sb 1.57383100 -0.48353300 -0.00001900

F 1.44682100 0.83780500 1.41805400

F 1.44676600 0.83783700 -1.41805500

N 3.80407000 0.76361000 0.00003300

H 3.74705800 1.36838300 -0.82181800

H 4.72265900 0.31890700 -0.00040700

H 3.74751900 1.36779700 0.82234000

Se-NH_3_

C -2.93575500 1.14686500 0.02551100

C -4.31038100 1.01777500 0.02271300

C -4.66359100 -0.41144900 0.01636400

C -3.50240000 -1.15726100 0.01525000

H -2.35221300 2.06762100 0.03098200

H -5.03004700 1.83963900 0.02489900

H -5.68161100 -0.80678600 0.01343700

H -3.40806400 -2.24432400 0.01120100

C -2.38289900 -0.20773200 0.02037700

C -1.06717200 -0.58752300 0.02172000

H -0.72860700 -1.62526800 0.02348200

O -0.10287500 0.39693500 0.03899400

Se 1.66946200 -0.00459400 -0.11522700

F 1.35335300 -1.85142300 0.06390500

F 1.91198200 1.81626900 0.05890600

N 4.39258300 -0.08801800 0.15055100

H 4.73421000 -0.61867900 0.95263200

H 4.62861000 0.89175200 0.31530400

H 4.93612700 -0.39477400 -0.65693300

Sb-NH_3_

C -3.40652500 -1.04903600 0.00001800

C -4.74619300 -0.69878700 -0.00000400

C -4.86230000 0.76156000 -0.00000900

C -3.59178400 1.30722600 0.00000100

H -2.98214200 -2.05339500 0.00002200

H -5.58903000 -1.39444800 -0.00001300

H -5.80189400 1.31867400 -0.00002100

H -3.32311600 2.36552000 0.00000500

C -2.64040400 0.19284300 0.00001300

C -1.27884900 0.35193300 0.00002400

H -0.83480300 1.35618300 0.00002400

O -0.43326000 -0.70099000 0.00004500

Sb 1.57383100 -0.48353300 -0.00001900

F 1.44682100 0.83780500 1.41805400

F 1.44676600 0.83783700 -1.41805500

N 3.80407000 0.76361000 0.00003300

H 3.74705800 1.36838300 -0.82181800

H 4.72265900 0.31890700 -0.00040700

H 3.74751900 1.36779700 0.82234000

Br-NH_3_

C 3.06918300 0.15117300 -1.06493400

C 4.41828100 0.13917300 -0.76293500

C 4.59044900 -0.05898800 0.68086500

C 3.34537200 -0.16854300 1.26707500

H 2.60766300 0.27921900 -2.04423600

H 5.23347300 0.25897900 -1.48030800

H 5.55034700 -0.10936300 1.19924200

H 3.11785300 -0.32144700 2.32357600

C 2.35250400 -0.04036000 0.19360500

C 1.00381600 -0.10432100 0.40211700

H 0.57144200 -0.25038900 1.39932300

O 0.13354500 0.00010100 -0.64715400

F -1.81408600 -1.89280900 -0.22883100

F -1.72331600 1.89917400 0.08745000

N -3.95094300 0.04287000 0.47087600

H -4.52464700 -0.38009900 -0.25960600

H -4.20997600 1.02444000 0.58382200

H -4.11935800 -0.45612100 1.34492000

Br -1.67084500 0.00250800 -0.12022700

I-NH_3_

C 3.42355900 0.01144800 -1.07289600

C 4.77595300 -0.00400800 -0.78523000

C 4.96159500 -0.03267300 0.66999400

C 3.72126900 -0.03489900 1.27668300

H 2.95130700 0.03343700 -2.05516200

H 5.58475900 0.00377200 -1.51967600

H 5.92658800 -0.04949400 1.18116200

H 3.50353200 -0.05331000 2.34617800

C 2.71805200 -0.00735400 0.20590400

C 1.37004600 -0.00183700 0.43196200

H 0.95664000 -0.01832900 1.44928900

O 0.48331400 0.02502700 -0.60528300

I -1.48579000 0.01956100 -0.09663700

F -1.65037800 2.03867000 0.00344100

F -1.65713000 -2.00834600 -0.07568900

N -3.90629400 -0.08864100 0.46109900

H -4.12817600 -1.08268700 0.55239200

H -4.49877100 0.31296800 -0.26721200

H -4.12674800 0.38017000 1.34108200

As-PH_3_

C -3.52213400 -0.97676900 -0.09380300

C -4.84090800 -0.56650500 -0.10060400

C -4.89341300 0.89948800 -0.01578800

C -3.60512400 1.38867400 0.04309500

H -3.14221700 -1.99720800 -0.14470300

H -5.71342700 -1.22135400 -0.15947700

H -5.80830200 1.49564800 -0.00221200

H -3.29144300 2.43172900 0.11170600

C -2.70196400 0.23117300 -0.00369200

C -1.34207100 0.32736000 0.03389900

H -0.82479000 1.29007400 0.10266200

O -0.55817800 -0.79944400 -0.01354500

As 1.23994600 -0.66547400 0.03228100

F 1.38374100 0.60574100 -1.18148600

F 1.33198800 0.46562700 1.37893700

P 4.53934000 0.40951500 -0.08370200

H 4.36960600 1.76596300 0.31273800

H 5.03156600 0.72185400 -1.38298000

H 5.82823600 0.26395000 0.50515800

Sb-PH_3_

C 3.78746300 -0.90106400 -0.06932200

C 5.09917800 -0.46288200 -0.06723600

C 5.11934400 1.00235500 0.00341200

C 3.81814100 1.46304600 0.04471900

H 3.43065700 -1.93026800 -0.11392900

H 5.98585600 -1.09993100 -0.11101800

H 6.02062500 1.61902100 0.02014400

H 3.48085400 2.49978000 0.09981600

C 2.94105700 0.28771300 0.00056500

C 1.57540500 0.35667500 0.02380500

H 1.05606200 1.32168300 0.07813200

O 0.80613600 -0.76603800 -0.01887400

Sb -1.18592900 -0.66742000 0.00855600

F -1.25353000 0.73087000 -1.32636900

F -1.22566800 0.60949800 1.45822300

P -4.23359600 0.75612500 -0.04996400

H -5.39031400 0.77207600 0.77825800

H -4.92118800 0.99842900 -1.27108000

H -3.85607400 2.10568800 0.18145700

Se-PH_3_

C -3.27809800 1.19911400 0.02714500

C -4.65614300 1.13592300 0.04288200

C -5.07822800 -0.27669200 0.02217300

C -3.95536300 -1.07724400 -0.00592100

H -2.65108600 2.09072500 0.03615000

H -5.33565100 1.99078800 0.06719800

H -6.11415200 -0.62217500 0.02855600

H -3.91293400 -2.16722300 -0.02638100

C -2.79151000 -0.18151600 -0.00367100

C -1.49610600 -0.62428700 -0.02633200

H -1.19466900 -1.67182300 -0.04552100

O -0.49335700 0.33261700 -0.02648700

Se 1.25608500 -0.11343300 -0.02262400

F 0.85309700 -1.94134300 0.02132900

F 1.50846500 1.70549100 -0.04308000

P 4.70289200 0.10068400 0.03764600

H 5.84745800 -0.74526400 -0.01181000

H 5.15227300 0.85555500 1.15828700

H 5.18399100 1.02582500 -0.93194100

Sb-PH_3_

C 3.78746300 -0.90106400 -0.06932200

C 5.09917800 -0.46288200 -0.06723600

C 5.11934400 1.00235500 0.00341200

C 3.81814100 1.46304600 0.04471900

H 3.43065700 -1.93026800 -0.11392900

H 5.98585600 -1.09993100 -0.11101800

H 6.02062500 1.61902100 0.02014400

H 3.48085400 2.49978000 0.09981600

C 2.94105700 0.28771300 0.00056500

C 1.57540500 0.35667500 0.02380500

H 1.05606200 1.32168300 0.07813200

O 0.80613600 -0.76603800 -0.01887400

Sb -1.18592900 -0.66742000 0.00855600

F -1.25353000 0.73087000 -1.32636900

F -1.22566800 0.60949800 1.45822300

P -4.23359600 0.75612500 -0.04996400

H -5.39031400 0.77207600 0.77825800

H -4.92118800 0.99842900 -1.27108000

H -3.85607400 2.10568800 0.18145700

Br-PH_3_

C 3.12629700 -0.97968200 -0.42255200

C 4.44045900 -1.04692700 -0.01079800

C 4.77143200 0.16363200 0.76132900

C 3.65704500 0.97138200 0.82713400

H 2.56394600 -1.71675000 -0.99548900

H 5.13192200 -1.86851200 -0.21097800

H 5.74424900 0.37481000 1.21060100

H 3.56295600 1.93942900 1.32158000

C 2.58694600 0.28296100 0.09096600

C 1.33275700 0.78958700 -0.09167000

H 0.98514200 1.73586800 0.33952500

O 0.47154700 0.15896500 -0.95733700

Br -1.26525400 0.04461000 -0.27865200

F -1.03393900 -1.80957300 -0.04084100

F -1.43821800 1.92036100 -0.49423200

P -4.04215600 -0.27901800 0.71724900

H -4.99386400 0.77514300 0.72275200

H -4.23391800 -0.70791200 2.05731800

H -4.85678000 -1.26268700 0.09668800

I-PH_3_

C 3.46024700 -1.04747000 -0.35595500

C 4.78131000 -1.08392900 0.04136600

C 5.10687200 0.15675700 0.76251100

C 3.98285300 0.95349200 0.81084300

H 2.90305300 -1.81154600 -0.89799400

H 5.48037400 -1.90376800 -0.13906800

H 6.08264000 0.39683000 1.19032400

H 3.88278000 1.93877600 1.26905900

C 2.91246300 0.22670300 0.11343600

C 1.64983700 0.71409200 -0.06242100

H 1.31709700 1.68209300 0.33067500

O 0.76189200 0.03383700 -0.86381600

I -1.14315100 0.07628300 -0.23151500

F -1.04067400 2.08278100 -0.43440800

F -1.01226700 -1.91608400 -0.06026500

P -4.22700500 -0.28626500 0.74116300

H -5.33440900 -0.17711700 -0.14193100

H -4.80350100 0.42839500 1.82526600

H -4.51610700 -1.59154400 1.22044400

As-AsH_3_

C 4.16072800 -0.90787100 -0.15124200

C 5.46359100 -0.45004000 -0.15712700

C 5.46463300 1.01275600 -0.01596900

C 4.16071200 1.45235600 0.07668700

H 3.81691300 -1.93868400 -0.23727700

H 6.35820000 -1.07000100 -0.25131400

H 6.35771300 1.64068800 0.01031000

H 3.81081100 2.47992600 0.18932900

C 3.29902600 0.26534200 -0.00480200

C 1.93743000 0.31027700 0.05214500

H 1.38544400 1.24924800 0.16451600

O 1.19500600 -0.84348500 -0.03049800

As -0.60387200 -0.77856000 0.04325800

F -0.72330000 0.29504000 1.43371500

F -0.81571600 0.53355800 -1.11671800

As -3.96680800 0.29900700 -0.06373000

H -4.38475600 0.70592100 -1.46181100

H -3.74893000 1.72379600 0.39935000

H -5.38858200 0.22793800 0.45532300

Sb-AsH_3_

C 4.33784800 -0.78420800 -0.14307800

C 5.62494300 -0.27887600 -0.13689300

C 5.57105200 1.18008600 0.01020400

C 4.24907200 1.56966900 0.09440100

H 4.03359500 -1.82691900 -0.23635400

H 6.54228800 -0.86563600 -0.22681100

H 6.43967800 1.84115300 0.04624700

H 3.86003000 2.58315400 0.20861200

C 3.43279200 0.35382100 0.00130000

C 2.06640200 0.34999800 0.04788300

H 1.49757300 1.28140300 0.16028500

O 1.35646000 -0.80994900 -0.04240100

Sb -0.63455000 -0.81593900 0.01463100

F -0.80198800 0.64189200 -1.24648300

F -0.72877600 0.38650300 1.52282300

As -3.81197600 0.54727400 -0.04982900

H -5.16936600 0.53472800 0.61950800

H -4.34404900 0.92330900 -1.41473700

H -3.46994800 1.96277500 0.35068400

Se-AsH_3_

C 3.78917200 -1.30858000 -0.02242300

C 5.16760000 -1.34788400 -0.04762500

C 5.69408600 0.02960000 -0.03027600

C 4.63455200 0.91148100 0.00559200

H 3.09757100 -2.15112300 -0.02598900

H 5.78133600 -2.25088400 -0.07611800

H 6.75264300 0.29705600 -0.04429600

H 4.67361600 2.00156500 0.02527500

C 3.40720300 0.10469600 0.01110900

C 2.14927400 0.64305800 0.04201000

H 1.92622300 1.70984900 0.06304700

O 1.07830800 -0.23812500 0.05698400

Se -0.63114900 0.33349700 0.00661700

F -0.09768400 2.12473200 -0.02478600

F -1.01386700 -1.46508100 0.06031700

As -4.07325100 -0.20979100 -0.01905300

H -5.52918200 0.20937700 -0.07211500

H -4.28163300 -1.19816400 1.10937500

H -4.21807300 -1.25956400 -1.10139400

Sb-AsH_3_

C -3.89724300 -1.46624500 -0.00016800

C -5.27363900 -1.57421600 -0.00017400

C -5.86637300 -0.22681500 0.00019500

C -4.85024700 0.70669100 0.00034800

H -3.16497300 -2.27380100 -0.00036900

H -5.84202300 -2.50711700 -0.00037800

H -6.93703300 -0.01160600 0.00030200

H -4.94316800 1.79392000 0.00059700

C -3.58416900 -0.03699700 0.00012700

C -2.35310400 0.56201000 0.00019000

H -2.20423000 1.64422300 0.00039300

O -1.23291000 -0.23805400 -0.00008900

Te 0.60424400 0.49003700 0.00000300

F 1.03215600 -1.44955600 -0.00027800

F -0.19149400 2.33627200 -0.00048600

As 4.09250900 -0.44947300 0.00013400

H 4.13848200 -1.47767900 -1.10851800

H 4.13849900 -1.47913200 1.10743700

H 5.58693300 -0.20071200 0.00047400

Br-AsH_3_

C 3.66865500 -0.98300900 -0.36224100

C 4.96020800 -1.06888500 0.11227400

C 5.27146200 0.13822700 0.89851600

C 4.16723900 0.96226000 0.91010400

H 3.12313600 -1.71252800 -0.96053600

H 5.64834000 -1.90086700 -0.05339900

H 6.22456700 0.33575600 1.39390000

H 4.06384400 1.93234300 1.39862600

C 3.12383700 0.28829100 0.12403000

C 1.88658700 0.81137600 -0.11960400

H 1.53024700 1.76154800 0.29593300

O 1.06116800 0.19321200 -1.02700200

Br -0.70068200 0.05318700 -0.40879700

F -0.46797900 -1.80279200 -0.19610800

F -0.88379600 1.93271300 -0.59578300

As -3.57400100 -0.15943700 0.48693600

H -4.65603500 0.56461600 -0.27708200

H -3.99219700 0.31340000 1.85839700

H -4.26732100 -1.49894900 0.54772500

I-AsH_3_

C 3.72245700 -1.19026200 -0.55118200

C 4.99986400 -1.52072400 -0.14864100

C 5.47781000 -0.52248300 0.82239100

C 4.49040800 0.41875500 1.01883400

H 3.08016700 -1.72641000 -1.24977300

H 5.57159300 -2.38862800 -0.48506000

H 6.45644200 -0.53665000 1.30700000

H 4.52173700 1.29227800 1.67196900

C 3.35620300 0.03297100 0.16683000

C 2.19431000 0.74238900 0.07370000

H 1.98843600 1.64818800 0.65625400

O 1.25983700 0.39419500 -0.87616100

I -0.64454800 0.50067100 -0.25303600

F -0.30649200 2.46775100 0.02826500

F -0.71058000 -1.48135000 -0.57303200

As -3.67081600 -0.63952200 0.41382600

H -4.13432700 -1.45944200 -0.76611600

H -5.05122900 -0.24574300 0.88779400

H -3.50620800 -1.80995500 1.35316800
